# Subcategorizing T1 Staging in Pancreatic Adenocarcinoma Predicts Survival in Patients Undergoing Resection: An Analysis of the National Cancer Database

**DOI:** 10.1089/pancan.2019.0017

**Published:** 2020-07-14

**Authors:** Mihir M. Shah, Rachel E. NeMoyer, Stephanie H. Greco, Chunxia Chen, Dirk F. Moore, Miral S. Grandhi, Russell C. Langan, Timothy J. Kennedy, Parisa Javidian, Salma K. Jabbour, H. Richard Alexander, David A. August, Darren R. Carpizo

**Affiliations:** ^1^ Division of Surgical Oncology, Department of Surgery, Emory University School of Medicine, Atlanta, Georgia, USA.; ^2^ Department of General Surgery, Rutgers Robert Wood Johnson Medical School, New Brunswick, New Jersey, USA.; ^3^ Division of Surgical Oncology, Rutgers Cancer Institute of New Jersey and Rutgers Robert Wood Johnson Medical School, New Brunswick, New Jersey, USA.; ^4^ Rutgers Cancer Institute of New Jersey, New Brunswick, New Jersey, USA.; ^5^ Department of Biostatistics, Rutgers School of Public Health, Piscataway, New Jersey, USA.; ^6^ Department of Pathology, Rutgers Robert Wood Johnson Medical School, New Brunswick, New Jersey, USA.; ^7^ Department of Radiation Oncology, Rutgers Cancer Institute of New Jersey and Rutgers Robert Wood Johnson Medical School, New Brunswick, New Jersey, USA.; ^8^ University of Rochester Medical Center, Department of Surgery, Rochester, New York, USA.

**Keywords:** AJCC staging, pancreatic adenocarcinoma, T1 subcategorization, T stage, N stage, National Cancer Database

## Abstract

**Purpose:** According to the American Joint Committee on Cancer (AJCC) 7th edition, T1 staging of pancreatic adenocarcinoma (PC) is defined as tumor limited to the pancreas, ≤2 cm. The AJCC 8th edition subcategorizes T1 staging into T1a (≤5 mm), T1b (≤1 cm), and T1c (≤2 cm) for PC despite the absence of supporting evidence. We sought to determine whether this new subcategorization has prognostic significance.

**Methods:** A retrospective review of patients undergoing definitive surgery for PC was performed by using the National Cancer Database (NCDB) from 2004 to 2014. Kaplan–Meier survival was computed for the subcategories. Multivariable analysis (MVA) was performed by using stepwise regression.

**Results:** The NCDB captured 41,552 stages I and II patients who underwent definitive surgery for PC in this 10-year period. A total of 2090 of these patients were pathological T1N0. The 5-year overall survival (OS) for patients with T1a (*n* = 319), T1b (*n* = 296), and T1c (*n* = 1309) PC was 68.8%, 57%, and 46.6%, respectively. This subcategorization lost significance on MVA and when focused on T1N1-2 patients. Recategorizing T stage into T1a (≤1 cm) and T1b (≤2 cm) resulted in statistical significance on MVA.

**Conclusion:** Subcategorization of the T1 stage into T1a, T1b, and T1c in resected PC does differentiate OS in patients with node-negative disease. We support the AJCC 8th edition T1 stage subcategorization, while understanding that it does not differentiate OS on MVA. When this is further subcategorized into T1a (≤1 cm) and T1b (≤2 cm), it predicts OS in resected, node-negative patients on MVA.

## Introduction

Accurate tumor nodes metastases (TNM) staging in oncology is essential not only for treatment recommendations but also for prognosis. Since 1977, the American Joint Committee on Cancer (AJCC) has developed staging guidelines for common solid organ tumors, including pancreatic cancer. The goal of the TNM staging system is to facilitate consistency in the description and reporting of neoplastic diseases to support treatment decisions for cancer patients and to help the evaluation of cancer outcomes more reliably.

Accurate preoperative determination of the AJCC stage of pancreatic adenocarcinoma (PC) currently requires assessment of the technical resectability of the tumor. This is unique to PC. For other gastrointestinal cancers, stages I–II usually indicate a tumor localized to the organ of origin, whereas stages III and IV indicate evidence of regional nodal and distant spread, respectively. The AJCC staging system is now in its 8th edition, and since the AJCC 6th edition, staging in pancreatic cancer was defined as follows: Stages I and II are defined by tumors that are resectable, stage III by tumors that are locally advanced and unresectable (regardless of nodal spread), and stage IV by distant spread. The 8th edition is somewhat different in that resectable N2 tumors are now stage III; nonetheless, this emphasizes the impact of resectability as a predictor of survival and de-emphasizes the use of T or N stage as predictors of survival.

The AJCC 8th edition was published in 2017 with an implementation date of January 1, 2018. Regarding PC, many changes have been made in the 8th edition of the AJCC staging system, including definitions of T and N stage^1^ (Table 1). There were many goals of these changes, including identifying T3 tumors in terms of size rather than extrapancreatic extension, allowing for easier reproducibility of T staging in practice, and a new N2 category was added following the pattern of other gastrointestinal tumor sites. According to the AJCC 7th edition, T1 stage of PC is defined as tumor limited to the pancreas that is 2 cm or less in greatest dimension.^2^ Notably, the AJCC 8th edition has subcategorized T1 staging into T1a (≤5 mm), T1b (<1 cm), and T1c (≤2 cm) for PC limited to the pancreas. There is currently no evidence that this subcategorization helps predict survival.

**Table 1.** Changes in Definition of T and N Stage (7th and 8th Editions) Based on American Joint Commission on Cancer Staging Manuals

T stage	AJCC 8th edition (2018)	AJCC 7th edition (2010–2017)
T stage		
T0	No evidence of tumor	No evidence of tumor
T1	Tumor ≤2 cm	Tumor limited to pancreas, ≤2 cm
T1a	Tumor ≤0.5 cm	
T1b	Tumor >0.5 and <1 cm	
T1c	Tumor 1–2 cm	
T2	Tumor >2 and ≤4 cm	Tumor limited to pancreas, >2 cm
T3	Tumor >4 cm	Extension into peripancreatic tissue (excluding arteries)
T4	Tumor involves celiac axis, SMA, and/or CHA	Tumor involves celiac axis or SMA
N stage		
Nx	Regional LNs not assessed	Regional LNs not assessed
N0	No regional LN metastases	No regional LN metastases
N1	Metastases in 1–3 regional LNs	Metastatic regional LNs
N2	Metastases in 4 or more regional LNs	N/A

Tumor size is in greatest dimension.

AJCC, American Joint Committee on Cancer; CHA, common hepatic artery; LN, lymph node; N/A, not applicable; SMA, superior mesenteric artery.

One of the defining features of PC is its proclivity for early dissemination. The overwhelming majority (>90%) of patients with pathologically defined localized pancreatic cancer undergo resection, experience recurrences, and eventually die of recurrent metastatic PC, which indicates that these patients harbor disseminated disease at the time of their resection.^3^ Further, studies involving genetically engineered mouse models of pancreatic cancer have shown that pancreatic ductal adenocarcinoma disseminates much earlier than expected (at the pre-invasive stage).^4^ Given this, we hypothesized that subcategorizing the T1 (2 cm) tumors into three subcategories based on differences in size of millimeters would not likely be predictive of survival, and would not support the AJCC 8th edition subcategorization. The purpose of this project was to determine whether the subcategorization of T1 staging has prognostic significance in resected patients.

## Methods

A retrospective review of patients undergoing definitive surgery for PC was performed by using the National Cancer Database (NCDB) from 2004 to 2014. The NCDB is a joint project of the Commission on Cancer of the American College of Surgeons and the American Cancer Society. The data used in the study were derived from a de-identified NCDB file. The American College of Surgeons and the Commission on Cancer have not verified and are not responsible for the analytic or statistical methodology employed, or the conclusions drawn from these data by the investigators. The Rutgers Institutional Review Board reviewed this study and exempted it for analyzing the NCDB. Histology codes on the basis of International Classification of Diseases for Oncology, 3rd edition (ICD-O-3.1) were used to select patients with PC. Of the 58 histology codes provided by NCDB, we used 22 codes associated with PC after consultation with our gastrointestinal pathologist (Table 2). Only patients with pathological T1 PC defined as 2 cm or less in greatest diameter were included in the initial analysis. Patients who did not survive 6 months from the day of diagnosis were excluded from this study. The reason for this is that 6 months is the period by which nearly all patients would have received either chemotherapy or radiation, if they were to receive that type of treatment at all. Using this 6-month period is known as the “landmark” method.^5^ To do otherwise would be to improperly introduce a time-dependent variable. We subsequently repeated the analysis to include the previously eliminated 491 patients who survived <6 months, and this did not alter the major results. Survival estimates were calculated by using the Kaplan–Meier method.^6^ Overall survival (OS) for node-negative patients was determined based on the T1 subcategories; it was similarly computed for node-positive patients, and for node-positive and node-negative patients combined. Multivariable proportional hazards analysis was performed by using stepwise regression for clinical variables that were associated with OS. The influence of T stage (T1–T3) and N stage (N0–N2) on OS was then determined for resectable pancreatic cancer by using the Concordance survival analysis.

**Table 2.** Histology (2090 Patients)

ICD-O-3.1 code	Histology	No. of patients
8012	Large cell carcinoma, NOS	1
8021	Carcinoma, anaplastic, NOS	2
8035	Carcinoma with osteoclast-like giant cells	4
8050	Papillary carcinoma, NOS	5
8140	Adenocarcinoma, NOS	1066
8144	Adenocarcinoma, intestinal type	2
8211	Tubular adenocarcinoma	8
8255	Adenocarcinoma with mixed subtypes	14
8260	Papillary adenocarcinoma, NOS	9
8290	Oxyphilic adenocarcinoma	1
8310	Clear cell adenocarcinoma, NOS	2
8450	Papillary cystadenocarcinoma, NOS	0
8453	Intraductal papillary-mucinous carcinoma, invasive	122
8470	Mucinous cystadenocarcinoma, NOS	32
8471	Papillary mucinous cystadenocarcinoma	1
8480	Mucinous adenocarcinoma	155
8481	Mucin-producing adenocarcinoma	5
8490	Signet ring cell carcinoma	4
8500	Intraductal carcinoma, NOS	646
8503	Intraductal papillary adenocarcinoma	10
8521	Infiltrating ductular carcinoma	1
8576	Hepatoid adenocarcinoma	0

ICD-O-3.1, International Classification of Diseases for Oncology, 3rd edition; NOS, not otherwise specified.

Survival curves were estimated by using the Kaplan–Meier method, and the effects of covariates on survival were assessed by using the Cox proportional hazards model.^7^ Statistical calculations were carried out by using SAS software version 9.4 (SAS Institute, 2018).

## Results

Using the NCDB, 41,552 stages I and II patients (T1–3, N0–1) were identified who underwent definitive surgery for PC. The median OS from the date of diagnosis was 20.1 months. Of these, 2090 patients had pT1N0 PC and survived at least 6 months from the day of diagnosis.

### Patient demographics, surgical and tumor characteristics, and adjuvant therapy characteristics

Of the 2090 patients, 47.5% were males. The majority of the patients had either one or no comorbidities (94.2%), moderately differentiated tumors (52.4%), and negative surgical margins (93.9%). Interestingly, despite the size of the tumor being ≤2 cm, 13.6% of the patients underwent preoperative radiation therapy (Tables 3 and 4).

**Table 3.** Patient Demographics, Surgical and Tumor Characteristics—Continuous Variables

Variable	N	Mean	SD	Minimum	Maximum
Age (years)	2090	65.6	10.75	21	90
Definitive surgery, days from Dx	2090	47.88	76.76	0	861
Regional nodes examined	2090	13.64	12.5	1	98

SD, standard deviation.

**Table 4.** Patient Demographics, Surgical and Tumor Characteristics—Categorial Variables

No.	Variable	Frequency	%
1.	Sex	2090	
	Male	992	47
	Female	1098	53
2.	Race	2061	
	White	1781	86
	Black	210	10
	Others	70	4
3.	Grade	1661	
	Well differentiated	401	24
	Moderately differentiated	871	52
	Poorly differentiated	357	22
	Undifferentiated/anaplastic	32	2
4.	Surgical margins	2066	
	Negative	1939	94
	Positive	127	6
5.	Radiation surgery sequence	2072	
	No RT	1436	69
	Preoperative RT	281	14
	Postoperative RT	355	17
6.	Systemic surgery sequence	1758	
	No systemic therapy	764	44
	Preoperative systemic therapy	296	17
	Postoperative systemic therapy	621	35
	Perioperative systemic therapy	77	4
7.	Charlson comorbidity score	2090	
	0	1408	67
	1	561	27
	2≥2	121	6
8.	Facility location	2065	
	North east	445	22
	South	775	37
	Midwest	515	25
	West	330	16
9.	Facility type	2065	
	Community	51	3
	Comprehensive community	535	26
	Academic/research	1266	61
	Integrated network	213	10
10.	Insurance status	2052	
	Not insured	39	2
	Private/managed care	834	41
	Medicaid	77	4
	Medicare	1071	52
	Other government	31	1

RT, radiation therapy.

### T1 subcategorization survival analysis

**FIG. 1.**
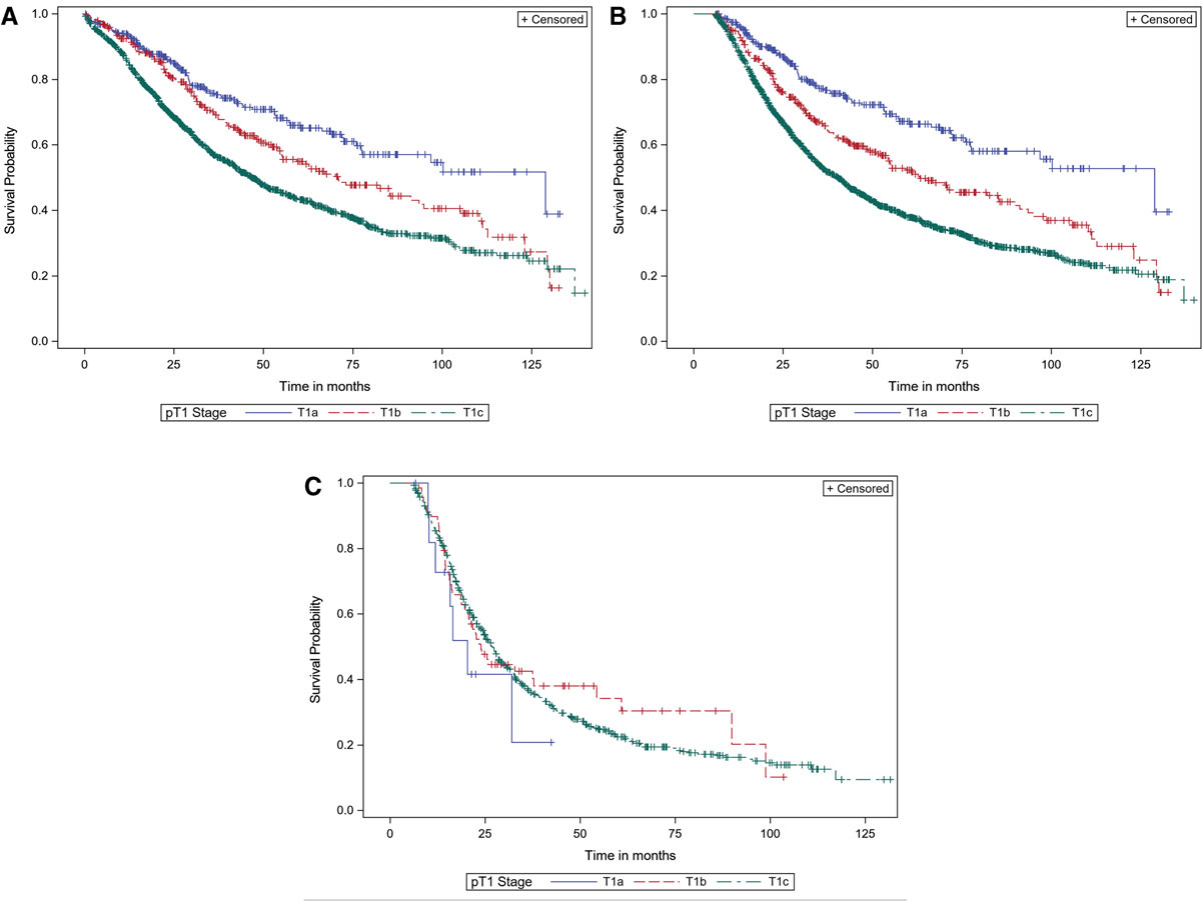
Overall survival for T1 pancreatic adenocarcinoma subcategorized by T1a, T1b, and T1c **(A.** Node-negative patients, **B.** Node-positive patients, **C.** Node-negative and -positive patients combined; Blue = T1a, Red = T1b, Green = T1c**)**.

When the T1 subcategory was used as a single variable in its association with OS (univariate analysis), the 5-year OS for patients with T1a (*n* = 319), T1b (*n* = 296), and T1c (*n* = 1309) PC was statistically significantly different (68.8%, 57.0%, and 46.6%, respectively; *p* < 0.0001; Fig. 1A). We then expanded the analysis to include node-positive patients (*n* = 816). These differences in OS remained statistically significant when node-positive and node-negative patients were combined (Fig. 1B), but they lost significance in node-positive patients alone (Fig. 1C).

We performed a multivariable proportional hazards analysis to control for potentially confounding clinical, pathologic, and demographic variables to determine whether T1 subcategorization remained predictive of OS. The variables that were predictive of OS are listed in Table 5. Increasing age, higher grade, and positive surgical margins were statistically significantly associated with worse OS. Interestingly, patients in the midwest, south, and west regions had a worse OS compared with patients in the northeast (Table 5). Note that T1 subcategorization was not statistically significant on this analysis. Given these findings, we sought to determine whether we could modify the T1 subcategorization such that it would maintain its prognostic value in multivariable analysis (MVA). We subcategorized T1 into two subclassifications, T1a (≤1 cm) and T1b (>1 and ≤2 cm), and this was statistically significant (hazard ratio = 0.791, *p* = 0.031)—T1a was associated with improved OS compared with T1b (Table 6). Increasing age, higher grade, and positive surgical margins remained independent predictors of worse OS. Interestingly, postoperative chemotherapy was significantly associated with improved OS compared with the lack of administration chemotherapy (*p* ≤ 0.0001).

**Table 5.** Multivariable Analyses: Stepwise Model Selection

Analysis of maximum likelihood estimates			
Parameter	DF	HR	p
Age, years			
≤50	1	0.533	0.0019
51–65	1	0.662	<0.0001
Facility location			
Midwest	1	1.328	0.048
South	1	1.653	0.0001
West	1	1.402	0.0354
Grade			
Moderately differentiated	1	1.877	<0.0001
Poorly differentiated	1	2.629	<0.0001
Undifferentiated/anaplastic	1	2.153	0.0547
Surgical margins			
Negative	1	0.559	0.0014
Radiation surgery sequence			
Preoperative RT	1	2.061	<0.0001
Postoperative RT	1	1.059	0.6304

For age, above 65 is the reference. For facility location, north east is the reference. For grade, well differentiated is the reference. For surgical margins, positive margin is the reference. For radiation surgery sequence, no RT is the reference.

DF, degree of freedom; HR, hazard ratio.

**Table 6.** Multivariable Analyses: Backward Selection

Analysis of maximum likelihood estimates			
Parameter	DF	HR	p
Age, years			
≤50	1	0.600	0.0071
51–65	1	0.699	0.0004
Grade			
Moderately differentiated	1	1.877	<0.0001
Poorly differentiated	1	2.629	<0.0001
Undifferentiated/anaplastic	1	2.153	0.0356
Surgical margins			
Negative	1	0.530	0.0003
Radiation surgery sequence			
Preoperative RT	1	2.108	0.0157
Postoperative RT	1	1.225	0.1278
Chemotherapy surgery sequence			
Preoperative CT	1	0.972	0.9201
Postoperative CT	1	0.764	0.0161
Perioperative CT	1	0.505	0.1180
T stage			
T1a (≤1 cm)	1	0.791	0.0312

For age, above 65 is the reference. For grade, well differentiated is the reference. For surgical margins, positive margin is the reference. For radiation surgery sequence, no RT is the reference. For chemotherapy surgery sequence, no chemotherapy is the reference. For T stage, T1b (>1 and ≤2 cm) is the reference.

CT, chemotherapy.

### AJCC 8th edition comparative analysis of T and N stage on survival

Our dataset indicated that the initial subcategorization of T stage (T1a, b, c) lost significance in patients with node-positive disease. This suggests that the N stage has greater impact on survival than the T stage in pancreatic cancer. This is not the case for other gastrointestinal cancers such as colorectal cancer, where the T stage has been shown to be of greater prognostic value.^8^ To evaluate this in PC, we used the NCDB pancreatic dataset to analyze stage T1–3 patients (*n* = 35,433). Results of Cox proportional hazards model for the effects of T stage and N stage on OS are presented in Tables 7 and 8.

**Table 7.** Shows the Stratification of 35,433 Patients with Pancreatic Cancer Based on the T Stage and N Stage

T and N stage	N0	N1	N2
T1	2108	725	84
T2	3124	2467	573
T3	7812	13396	5144

**Table 8.** Demonstrates the Cox Model for Predicting Overall Survival Using the T Stage and N Stage (Nodal Status)

Variable	Log HR	HR	95% CI	p
N1	0.443	1.557	1.513–1.602	2 × 10^−6^
N2	0.701	2.016	1.943–2.092	2 × 10^−6^
T2	0.367	1.444	1.363–1.530	2 × 10^−6^
T3	0.506	1.659	1.573–1.749	2 × 10^−6^

N0 = negative nodes; N1 = 1–3 positive nodes; N2 = 4 or more positive nodes; T1 = size ≤2 cm in greatest dimension; T2 = size >2 and ≤4 cm in greatest dimension; T3 = size >4 cm in greatest dimension; For node status, N0 is the reference; For T stage, T1 is the reference; N0 = negative nodes; N1 = 1–3 positive nodes; N2 = 4 or more positive nodes; T1 = size ≤2 cm in greatest dimension; T2 = size >2 and ≤4 cm in greatest dimension; T3 = size >4 cm in greatest dimension.

CI, confidence interval.

**FIG. 2.**
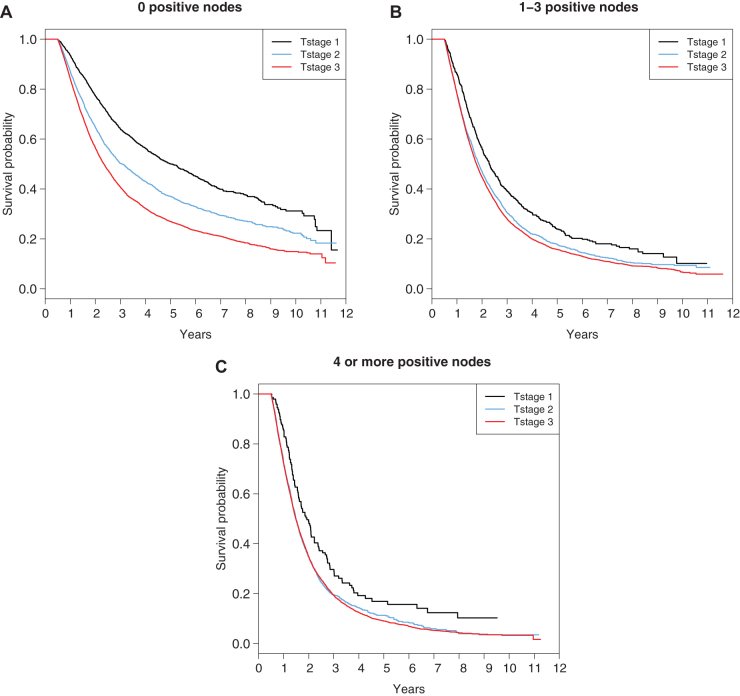
Overall survival curves for a different T stage for each N stage in pancreatic adenocarcinoma **(A.** N0, **B.** N1 {1–3 positive nodes}, **C.** N2 {4 or more positive nodes}**)**.

Judging from the magnitudes of the log-hazard ratios, nodal status is more influential than the T stage in predicting survival, although both are highly significant (*p* < 0.0001). Figure 2A–C demonstrates the survival plot based on T stage for each N stage (N0, N1, and N2, respectively).

Another way of judging influence on survival is the “Concordance,” which in survival analysis measures the ability to predict which patients will die sooner. Technically, it is the proportion of pairs of cases in which the case with the higher risk predictor had an event before the case with the lower risk predictor. Judging by this, nodal involvement had a concordance of 0.578, which is higher than T stage (0.543). It is not clear, however, that this difference is clinically relevant.

## Discussion

Patients with early stage PC who undergo resection almost uniformly suffer recurrence and eventually die from their disease, with 40% 5-year survival for stage IA PC (T1N0).^9^ This is generally believed to be due to the fact that the disease disseminates early in its tumor formation. Given this aggressive disease biology, it is surprising that in node-negative patients a difference of 5 mm in tumor size (on univariate analysis) and 10 mm (on multivariate analysis) does, indeed, predict survival. If tumor size correlates with burden of disseminated tumor cells, then perhaps this can be explained by the fact that tumor volume is proportional to the cube of tumor diameter, and so there are ∼10-fold million more cells in a 10-mm tumor as compared with a 5-mm tumor.^10^

As with many solid malignancies, including PC, the total diameter of the tumor has been linked strongly to OS. In the 7th edition, T3 tumors were not defined by their diameter but rather by their relationship to peripancreatic tissue. Our data indicate that the 8th edition change in T stage (by diameter) is predictive and is an improvement over the 7th edition. This finding has also been demonstrated in a recent retrospective analysis combining data from three centers.^1,11^ Interestingly, the overall T stage in AJCC 8th edition is very similar to the AJCC 1st edition in 1977—T3 is defined by size rather than extension of the tumor (>4 vs. >6 cm, respectively). The evolution of the T and N staging system in pancreatic cancer over all eight editions of the AJCC system is shown in Tables 9 and 10. T1N0 patients clearly have the best survival among resected patients (5-year survival = 50%, based on our results). However, it is worthwhile to mention that small-intraductal papillary mucinous neoplasm associated PC (<2 cm) have a reported 5-year survival of 59%, with superior survival for colloid carcinoma (95% 2-year survival).^12^

**Table 9.** Changes in Definition of T and N Stages Based on American Joint Committee on Cancer Staging Manuals

8th Edition (2018)	7th Edition (2010–2017)	6th Edition (2003–2009)	5th Edition (1998–2002)	4th Edition (1993–1997)	3rd Edition (1989–1992)	2nd Edition (1985–1988)	1st Edition (<1985)
T stage							
T1 (≤2 cm)				T1a (≤2 cm)		T1 (limited to pancreas)	T1 (<2 cm)
T2 (>2 and ≤4 cm)	T2 (>2 cm)			T1b (>2 cm)			T2 (2–6 cm)
T3 (>4 cm)	T3 (EPE)		T3^a^	T2^a^		T2^c^	T3 (>6 cm)
T4 (celiac, SMA or CHA)	T4 (celiac or SMA)		T4^b^	T3^b^		T3 (further EPE)	T4 (EPE)
N stage							
N1 (1–3 LN)	N1 (LN)		N1a (single LN)	N1 (LN)			N1 (1 LN group at laparotomy)
N2 (≥4 LN)			N1b (multiple LN)				N2 (2 or more LN groups at laparotomy)
							N3 (clinical evidence of LN; no laparotomy)
							N4 (Juxta-LN)

^a^ Extends directly into duodenum, bile duct, or peripancreatic tissues.

^b^ Extends directly into stomach, spleen, colon, or adjacent large vessels.

^c^ Limited EPE to duodenum, bile ducts, or stomach, still possibly permitting tumor resection.

EPE, extrapancreatic direct extension; LN, regional lymph node.

**Table 10.** Changes in Definition of TNM Stages Based on AJCC Staging Manuals

8th Edition (2018)	7th Edition (2010–2017)	6th Edition (2003–2009)	5th Edition (1998–2002)	4th Edition (1993–1997)	3rd Edition (1989–1992)	2nd Edition (1985–1988)	1st Edition (<1985)
Stage I							
A: T1 N0 M0 B: T2 N0 M0 T1a, T1b, T1c	A: T1 N0 M0 B: T2 N0 M0		T1-2 N0 M0				NR
Stage II							
A: T3 N0 M0 B: T1-3 N1 M0			T3 N0 M0				NR
Stage III							
T1-3 N2 M0 T4 N0-2 M0	T4 N0-1 M0		T1-3 N1 M0				NR
Stage IV							
T1-4 N0-2 M1	T1-4 N0-1 M1		A: T4 N0-1 M0 B: T1-4 N0-1 M1	T1-3 N0-1 M1			NR

NR, not recommended.

Although changes are necessary to the AJCC as new diagnostic tools and better understanding of disease processes occur, frequent changes to the AJCC staging system without data to support the changes are, indeed, predictive of survival and can lead to problems.^13^ A major strength of this study is the large size of the NCDB, which, like the Surveillance, Epidemiology, and End Results Program database, is ideally suited to provide validation to new changes in TNM staging systems. The data presented here are powered by large numbers and supported by strong statistical analysis, but nevertheless are subject to several limitations. First, the retrospective nature of these data introduces the known challenges of observational studies, including limitations on survival data secondary to loss of follow-up as well as selection bias. Although MVA was performed to control for known confounding variables, we cannot reliably reduce unmeasured factors that may account for imbalances between the groups. Finally, our study was limited by potential coding errors, missing data, and the absence of several variables within the NCDB, including disease-specific survival, type of chemotherapy, and duration of chemotherapy.

In conclusion, our data indicate that subcategorization of the T1 stage into T1a, T1b, and T1c is, indeed, predictive of OS in node-negative patients, supporting the AJCC 8th edition and providing evidence for the subcategorization. We understand that this does not hold true on MVA. However, we note that subcategorizing T1 into two subcategories: T1a (≤1 cm) and T1b (≤2 cm) significantly stratify patients by OS on MVA.

## Abbreviations Used

AJCC: American Joint Committee on Cancer
CHA: common hepatic artery
CI: confidence interval
CT: chemotherapy
DF: degree of freedom
EPE: extrapancreatic direct extension
HR: hazard ratio
ICD-O-3.1: International Classification of Diseases for Oncology, 3rd edition
LN: lymph node
MVA: multivariable analysis
NCDB: National Cancer Database
NR: not recommended
OS: overall survival
PC: pancreatic adenocarcinoma
RT: radiation therapy
SD: standard deviation
SMA: superior mesenteric artery
